# A randomized controlled trial of exercise during pregnancy on maternal and neonatal outcomes: results from the PAMELA study

**DOI:** 10.1186/s12966-017-0632-6

**Published:** 2017-12-22

**Authors:** Shana Ginar da Silva, Pedro Curi Hallal, Marlos Rodrigues Domingues, Andréa Dâmaso Bertoldi, Mariângela Freitas da Silveira, Diego Bassani, Inácio Crochemore Mohnsam da Silva, Bruna Gonçalves Cordeiro da Silva, Carolina de Vargas Nunes Coll, Kelly Evenson

**Affiliations:** 1 Postgraduate Program in Epidemiology, Federal University of Pelotas, Rua Marechal Deodoro, 1160-3° piso, CEP: 96020-220, Bairro Centro, Pelotas, Rio Grande do Sul Brazil; 2 Department of Epidemiology, Gillings School of Global Public Health, University of North Carolina – Chapel Hill, Chapel Hill, NC USA; 3 Postgraduate Program in Physical Education, Federal University of Pelotas, Pelotas, Brazil; 4 Department of Paediatrics, Faculty of Medicine, Centre for Global Child Health, University of Toronto; King’s College Circle, The Hospital for Sick Children, Toronto, Canada

**Keywords:** Exercise, Randomized controlled trial, Pregnant woman, Maternal-child health, Physical activity, Intervention studies

## Abstract

**Background:**

Women are encouraged to be physically active during pregnancy. Despite available evidence supporting antenatal physical activity to bring health benefits for both the mother and child, the most effective way to prevent some maternal and fetal outcomes is still unclear. The purpose of this study was to evaluate the efficacy of an exercise intervention to prevent negative maternal and newborn health outcomes.

**Methods:**

A randomized controlled trial (RCT) nested into the 2015 Pelotas (Brazil) Birth Cohort Study was carried-out with 639 healthy pregnant women, 213 in the intervention group (IG) and 426 in the control (CG) group. An exercise-based intervention was conducted three times/week for 16 weeks from 16-20 to 32-36 weeks’ gestation. The main outcomes were preterm birth and pre-eclampsia. Gestational age was calculated based on several parameters, including routine ultrassounds and/or last menstrual period and categorized as < 37 weeks and ≥ 37 weeks for evaluation of preterm birth. Pre-eclampsia was self-reported. Secondary outcomes were gestational weight gain, gestational diabetes, birth weight, infant length, and head circumference. Analyses were performed by intention-to-treat (ITT) and per protocol (70% of the 48 planned exercise sessions). Odds ratio were derived using unconditional logistic regression.

**Results:**

The IG and CG did not differ at baseline regarding their mean age (27.2 years ± 5.3 vs. 27.1 years ± 5.7) and mean pre-pregnancy body mass index (25.1 ± 3.9 vs. 25.2 ± 4.1 kg/m^2^). The mean adherence to the exercise intervention was 27 ± 17.2 sessions (out of a potential 48) with 40.4% attending > = 70% of the recommended exercise sessions. A total of 594 participants (IG:198; CG: 396) were included in the ITT and 479 (IG: 83; CG: 396) were included in the per protocol analyses. There were no significant differences in the incidence of preterm birth and pre-eclampsia between groups in the ITT and per protocol analysis. There were also no differences between the two groups in mean gestational weight gain, gestational diabetes, birth weight, infant length, and head circumference.

**Conclusions:**

While the RCT did not support the benefits of exercise performed during pregnancy on preeclampsia and preterm birth, the exercise program also did not present adverse impacts on newborn health. Our findings may contribute to promote intervention strategies that motivate health providers to encourage pregnant women to be more physically active.

**Trial registration:**

Clinicaltrials.gov identifier: NCT02148965, registered on 22 May 2014.

**Electronic supplementary material:**

The online version of this article (10.1186/s12966-017-0632-6) contains supplementary material, which is available to authorized users.

## Background

Women with uncomplicated pregnancies should be physically active during pregnancy. A 2014 review of physical activity guidelines during pregnancy around the world indicated universal support of moderate-intensity physical activity during uncomplicated pregnancy [1]. The American College of Obstetricians and Gynecologists (ACOG) recommends that women with uncomplicated pregnancies should engage in moderate intensity exercise 20–30 min/day on most or all days of the week during pregnancy [2]. The United States Department of Health and Human Services recommends that women with an apparently healthy pregnancy should accumulate at least 150 min/ week of moderate-intensity aerobic activity during both pregnancy and postpartum [3]. According to the World Health Organization (WHO), counselling about keeping physically active during pregnancy is recommended for pregnant women to stay healthy and to prevent excessive gestational weight gain [4]. Despite available evidence supporting the promotion of antenatal physical activity to bring health benefits for both mother and child, the most effective way to prevent some maternal and fetal outcomes is still unclear. Initial studies in the area were concerned about the potential risks of exercise on newborn health [5]. These hypotheses have not been proven over time but concerns about the safety of exercise during pregnancy seem to remain.

Previous reviews and meta-analyses summarized the associations of physical activity during pregnancy with specific maternal and child health outcomes [6, 7]. Observational studies showed positive associations between leisure-time physical activity (LTPA) and maternal–child health [7–10], while most randomized controlled trials (RCTs) reported no associations [6, 11]. Nevertheless, a recent meta-analysis including only RCTs indicated exercise programs during pregnancy prevented excessive weight gain, gestational diabetes, and newborn’s large-for-gestational age [12]. No effects of exercise during pregnancy on pre-eclampsia, preterm birth, or birth weight were observed [12].

Key limitations of previous RCTs include small sample size, self-selection, high dropout rates, and low adherence to the exercise protocol [12]. In addition, many interventions on physical activity and maternal-child-health are based only on counseling strategies and information about physical activity during pregnancy and postpartum, and do not include exercise sessions [13]. In 2014, a RCT nested in the 2015 Pelotas (Brazil) birth cohort study was planned with a number of strategies to address the limitations identified in prior studies. The main reason for conducting this trial in Brazil is the fact that LTPA among Brazilian women is associated with socioeconomic factors and other characteristics not easily controlled during statistical analyses in merely observational studies. Moreover, few experimental studies have been carried out to evaluate these associations in low or middle income countries with large population-based samples [14]. Therefore, the purpose of this study was to evaluate the efficacy of a supervised exercise-based intervention performed from 16-20 to 32-36 weeks’ gestation to prevent maternal (gestational diabetes, excessive weight gain, and pre-eclampsia) and newborn (preterm birth, and low birth weight) negative health outcomes assessed in a Brazilian population-based cohort study.

## Methods

### Trial design and setting

The PAMELA (Physical Activity for Mothers Enrolled in Longitudinal Analysis) trial is a randomized controlled trial nested into the 2015 Pelotas (Brazil) Birth Cohort Study. Eligible pregnant women were sampled from the antenatal phase of the 2015 Cohort, a population-based cohort study of all births from mothers living in the urban area of the city of Pelotas, Brazil. The 2015 Pelotas Birth Cohort Study recruited pregnant women from all health facilities offering antenatal care (public and private) including clinical laboratories, ultrasound clinics, basic health units, hospitals, clinics/polyclinics, colleges and private doctor offices in the city of Pelotas. Pregnant women with an expected delivery date from January 1^st^ 2015 to 31^st^ December 2015 were eligible for the cohort. Participants of the antenatal phase of the cohort study were recruited to enroll the RCT prior to 20 weeks of gestation starting in April 2014 and ending in October 2015, by a standard phone contact. In order to achieve the required sample size, recruitment was extended until March 2016, using the same eligibility criteria, and recruited an additional sample of 41 pregnant women.

The trial protocol and the 2015 Pelotas Birth Cohort Study were submitted to the Physical Education School and Medical School Ethics Committee and were approved under the numbers 649.244 and 522.064, respectively. The study is also registered on the Clinicaltrials.gov website under the registry number NCT02148965. Details on the trial design, recruitment and protocol can be found elsewhere [14]. This trial is reported according to the Consolidated Standards of Reporting Trials (CONSORT) [15] and the 16-item internationally endorsed Consensus on Exercise Reporting Template (CERT) [16].

### Inclusion and exclusion criteria

Women whose pregnancy exercise levels did not include self-reported participation in an exercise program (LTPA > 150 min/week), 18 years or older and living in the urban area of the city of Pelotas, Rio Grande do Sul State, Brazil were eligible for the trial. Exclusion criteria were self-reported hypertension, cardiovascular disease, or diabetes diagnosed before pregnancy, history of miscarriage or preterm birth, in vitro fertilization in the current pregnancy, twin pregnancy confirmed by ultrasound, persistent bleeding in the current pregnancy, body mass index (BMI) > 35 kg/m^2^, and heavy smoker (> 20 cigarettes a day).

### Randomization

Eligible women provided written informed consent before taking part in the study and completed a baseline assessment at the Epidemiological Research Center of the Federal University of Pelotas. Participants were then assigned to either an exercise or control group using a computerized random-number generator. The randomization process occurred in blocks of nine pregnant women. Each block resulted in the allocation of three women for the intervention and six women for the control group, ensuring a recruitment balance of 1:2 throughout the study. We used 2 controls to 1 case in order to increase precision and statistical power of detecting a statistically significant difference if such a difference exists [15]. We chose to increase the number of individuals in the control group instead of individuals in the intervention group given the high costs associated with the intervention.

The nature of this trial meant that participants and staff were not masked to the type of intervention. However, the principal researcher was not involved in the exercise training and analyses were performed blinded for group allocation. Also, the staff involved with exercise intervention or outcome assessments had no influence on the randomization procedure. The assessors of the primary study outcomes were blinded.

### Intervention

The exercise training program started between 16 and 20 weeks’ gestation and was continued for at least 16 weeks [14]. Women in the intervention group received a structured, individually supervised, moderate-intensity exercise program for 1 hour 3 days/week planned according to the ACOG recommendations [2]. Each session involved warm-up, aerobic activities (treadmill or stationary bike), strength training (dumbbells, machines or elastic bands), and stretching exercises. The exercise intensity was measured according to each woman’s perceived effort (within the range of 12 to 14 on the Borg Scale) [17]. A mean of 48 training sessions were planned for each participant. The training sessions were grouped into three stages. The first stage (week 1 to 4) began with 5 min warm-up period, 15 min aerobic exercise, 35 min strength training/floor exercises (sets: 3 × 12 repetitions), and 5 min stretching. The second stage (week 5 to 10) started with 5 min warm-up period, 20 min aerobic exercise, 30 min strength training/floor exercises (sets: 3 × 10 repetitions), and 5 min stretching. Lastly, the third stage (11 to 16) began with 5 min warm-up period, 25 min aerobic exercise, 25 min strength training/floor exercises (sets: 3 × 8 repetitions), and 5 min stretching [18, 19].

Sessions were guided by a team of five trained physical education professionals. In order to offer personalized supervision, each shift counted on the presence of two physical education professionals and a maximum of six pregnant women per hour. The intervention program was performed at Federal University of Pelotas at the gym of the Physical Education School.

### Control group

Women allocated to the control group received standard antenatal care and were encouraged to continue their normal daily activities. They received the same assessments as the intervention group and were followed by the 2015 Pelotas (Brazil) Birth Cohort Study.

### Strategies to promote adherence

To reduce dropout and to increase adherence to the exercise training program, participants were informed of the importance to attend all sessions [14]. Adherence to the exercise sessions was controlled by the instructors, and registered in a personal training diary. Strategies such as door-to-door transportation and a kit, containing a t-shirt, running tights, and running shoes, were offered to participants to improve adherence. Both groups received study t-shirts and laboratory results around 10 days after baseline data collection. To be considered adherent to the intervention, women must have attended at least 34 of 48 (70%) of the prescribed workout sessions. Adherence criteria was verified by checking the percentage of supervised exercise sessions completed by each participant, defined as the number of sessions attended from the start of the trial up to the moment that participants decided to stop (before or after 16-weeks), divided by the minimum number of supervised exercise sessions prescribed.

## Assessesments

### Baseline measures

After enrollment, women were invited to visit the Epidemiological Research Center to collect baseline data. The baseline data was collected prior to 20 weeks’ gestation and included blood and urine sampling, anthropometry (weight and height), blood pressure measurement, lung function, and back pain tests. Blood pressure (systolic and diastolic) was measured twice after 2 min of seated rest using a sphygmomanometer model UM080. The same assessments were repeated at eight and 16 weeks after baseline. Maternal and neonatal outcomes were collected at the hospital up to 48 h after delivery via face-to-face interviews by trained staff.

### Primary outcomes

The primary outcomes were preterm birth and pre-eclampsia. Gestational age at birth was calculated based on a series of information collected in antenatal and perinatal study as followings: (1) data on the last menstrual period (LMP) were collected (on the pregnant woman’s prenatal care card) and/or by self-report; (2) gestational age was also collected through the ultrasound performed in the 1st and 2nd trimesters of gestation. The final variable of gestational age was estimated by an algorithm that considered all information collected, as well as the plausibility on estimates based on birth weight, length and head circunference, according to the Fetal and Neonatal Growth Curves for the twenty-first Century [20]. Births were categorized as preterm when the gestational age at birth was < 37 weeks and term when the gestational age at birth was ≥ 37 weeks [21]. Pre-eclampsia was defined by self-report within 48 h after delivery using the question, “Do you have eclampsia or pre-eclampsia?” Mothers answered “yes” or “no.”

### Secondary outcomes

The secondary outcomes were gestational weight gain, gestational diabetes mellitus (GDM), birth weight, small and large-for-gestational age, and other offspring characteristics (infant length, and head circumference). Current weight was measured to the nearest 0.1 kg on electronic TANITA (BF-680 W, model UM080; Tanita, Tokyo, Japan) scales at baseline, and both 8 and 16 weeks later. Height was measured using a tape measure fixed to the wall and a moveable head board at baseline only. Gestational weight gain was calculated in two ways: (1) using weight measured at baseline subtracted by weight measured at the last visit to the clinic, 16 weeks after baseline; and (2) assessed following the 2009 Institute of Medicine (IOM) recommendations [22] based on pre-pregnancy BMI and total gestational weight gain measured by self-reported and collected at the hospital up to 48 h after delivery. Pre-pregnancy BMI was calculated by dividing the weight by the squared height (kg/m^2^) and categories were defined according to WHO [23]. Recommended weight gain during pregnancy for underweight, normal weight, overweight, and obese women were 12.5 to 18 kg, 11.5 to 16 kg 7 to 11.5 kg, and 5 to 9 kg, respectively.

GDM was self-reported and evaluated during the hospital stay at delivery. Birth weight was collected from medical records at the hospital and categorized as low birth weight < 2500 g, normal birth weight ≥ 2500 g and macrosomia > 4000 g [24]. Small-for-gestational age and large-for-gestational age were defined according Intergrowth 21-st newborn standard [25]. Length and head circumference were measured at the hospital up to 48 h after delivery by trained staff.

### Covariates

At the first visit (up to 20 weeks’ gestation) during antenatal phase of the birth cohort study, the mothers were interviewed face-to-face by trained staff about maternal age, education, pre-pregnancy weight, marital status, employment during pregnancy, skin color, and current smoking. Forty-one pregnant women were recruited using a convenience sample after the prenatal care follow-up. These women did not answer the complete questionnaire and were not included in prenatal care measures.

### Sample size calculation

Sample size calculations have been described in detail elsewhere [14] with the study powered to detect differences for the two main outcomes. Based on statistical power of 80% and a level of significance set at 5%, we estimated that 213 women would be necessary for the intervention group. The intervention:control ratio was 1:2, therefore 426 women were included as the control group.

### Statistical analysis

Statistical analyses were conducted primarily on intention-to-treat (ITT) basis and per protocol analyses were also performed including only those adhering to the protocol (at least 34/48 (70%) sessions attended). In addition, a sensitivity analysis to account for the effect of protocol deviation [26] was performed based on adherence to at least 48 sessions (100% of exercise sessions). Baseline characteristics were presented using descriptive statistics to compare both groups. Group-mean differences by covariates were analyzed using the Student’s t-test (mean, SD) for continuous variables or the chi-squared test for categorical variables (*n*, %). Normality of continuous variables were checked graphically using histograms and by mean, median, skewness, and kurtosis parameters. All continuous variables presented symmetric distribution. The offspring characteristics (birth weight, lengh and head circunference) were also analyzed by Z-scores. Due to the similarity of the results between the Z-scores and mean values of the offspring characteristics, we chose to present the results in means and standard deviations for a better interpretation. Odds ratios were derived using unconditional logistic regression. Statistical significance was assessed using 95% confidence intervals. All the analyses were performed using the software Stata version 12.1 (StataCorp, College Station, Texas, US).

## Results

**Fig. 1:**
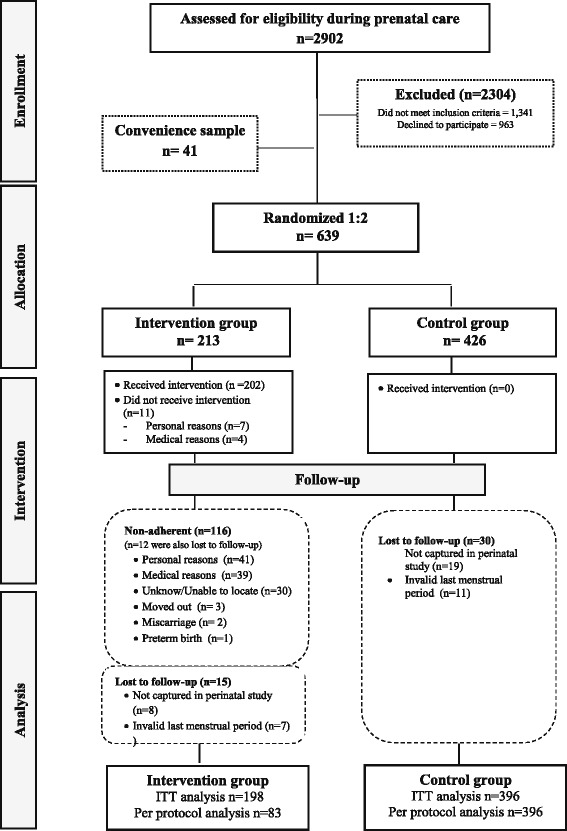
Flow diagram of the PAMELA study following the CONSORT guidelines. ITT: Intention-to-threat analysis. 4 women reached out the exercise program adherence criteria after the 16th week of the intervention

From a total of 2902 assessed for eligibility, 1341 pregnant women did not meet inclusion criteria and 963 declined to participate (Fig. 1). A total of 639 were randomized to either the intervention (*n* = 213) or the control group (*n* = 426). During the course of the study, 116 women from the intervention group were non-adherent because of personal reasons (*n* = 41), medical reasons (*n* = 39), unknown reasons/unable to locate (*n* = 30), moved out (*n* = 3), miscarriage (*n* = 2) and preterm birth (*n* = 1). Fourteen women in the intervention and 30 in the control group were lost to follow-up because they were not captured in the perinatal study (*n* = 8, intervention; *n* = 19 control) and 6 women had invalid data for last menstrual period in the intervention group, compared to 11 in the control group. A total of 594 participants were included in the ITT (198 in the intervention and 396 in the control groups) and 479 (83 in the intervention and 396 in the control groups) were included in the per protocol analyses analysis (Figure 1).

There were no statistically significant differences in the baseline and prenatal characteristics between intervention and control groups (Table 1). For example, the intervention and control groups did not differ at baseline regarding their mean age (27.2 years ± 5.3 vs. 27.1 years ± 5.7) and mean pre-pregnancy BMI (25.1 kg/m^2^ ± 3.9 vs. 25.2 kg/m^2^ ± 4.1). The samples used in ITT and per protocol analyses also did not present differences for key maternal characteristics evaluated at baseline (Additional file 1: Table S1).

**Table 1:** Maternal characteristics in the intervention and control groups, PAMELA study

	Intervention (*n* = 213)	Control (*n* = 426)	*p*
*Baseline measures (16-20 weeks gestation)*			
Maternal age (years)	27.2 ± 5.3	27.1 ± 5.7	0.83
Gestational age (weeks)	16.4 ± 1.6	16.4 ± 1.5	0.74
Weight (kg)	69.1 ± 12.8	69.4 ± 13.0	0.77
Height (cm)	161 ± 6.6	161 ± 6.0	0.29
Blood systolic pressure (mmHg)	111.6 ± 10.2	112.1 ± 10.4	0.58
Blood diastolic pressure (mmHg)	68.8 ± 7.9	69.3 ± 7.3	0.16
Proteinuria (mg/DL)^ǂ^	12.2 ± 5.9	12.4 ± 6.7	0.67
Fasting glycemia (mg/DL)	82.6 ± 8.5	82.4 ± 7.9	0.80
*Prenatal care measures (up to 20 weeks gestation)*	Intervention (*n* = 199)	Control (*n* = 399)	*p*
Schooling (years)^b^	12.4 ± 3.7	11.9 ± 3.5	0.09
Pre-pregnancy body mass index (kg/m^2^)^a^	25.1 ± 3.9	25.2 ± 4.1	0.94
Pre-pregnancy body mass index (≥ 25 kg/m^2^)^a^	81 (44.3)	169 (46.1)	0.69
Nulliparity			
Yes	124 (64.9)	251 (66.1)	0.73
Skin color^b^			
White	147 (73.9)	308 (77.6)	0.12
Marital Status			
Living with a partner	170 (85.4)	345 (86.5)	0.73
Smoking during pregnancy^c^			
Yes	13 (6.9)	16 (4.4)	0.21
Employment during pregnancy			
Yes	109 (58.3)	239 (62.3)	0.33

Data are expressed as means with standard deviation (SD) and *n*. (%). *p* > .05

No statistically significant differences between groups. Group-mean differences according covariates were analyzed by the Student’s t-test (mean, SD) or chi-squared test (*n*, %). ^ǂ^1 missing;^a^48 missing; ^b^1 missing; ^c^ 42 missing; ^c^ 29 missing

The mean attendance to the intervention program was 27 sessions (± 17.2) with 86 of 213 women (40.4%) having > = 70% adherence (at least 34 sessions). When analyzing women with 48 sessions or more, we found that only 23 of 213 women (11%) attended all planned sessions (48 sessions). Women who were adherent to the intervention were older, had higher schooling, and did not smoking during pregnancy (Additional file 2: Table S2).

The mean gestational age did not differ between intervention and control groups using ITT or per protocol analysis (> = 70% or 100%) (Table 2). Preterm births also did not differ between intervention and control groups using ITT or per protocol analysis (> = 70% or 100%).

**Table 2:** Comparisons of gestational age and preterm birth between control and exercise groups, PAMELA study

*Intention-to-treat analysis*	Intervention (*n*=198^a^)	Control (*n*=396^a^)	*p*	
	*n* (%)		0.73	OR (95%CI)
Preterm birth				
< 37 weeks	26 (13.1)	48 (12.1)		1.1 (0.7-1.8)
≥ 37 weeks	172 (86.9)	348 (87.9)		–
	mean (SD)			MD (95%CI)
Gestational age at delivery (weeks)	38.5 ± 2.1	38.7 ± 1.8	0.17	0.2 (−0.1-0.6)
* Per protocol analysis (> = 70% exercise sessions)*	*n* = 83	*n* = 396		
	*n* (%)		0.52	OR (95%CI)
Preterm birth				
< 37 weeks	8 (9.6)	48 (12.1)		0.8 (0.4-1.7)
≥ 37 weeks	75 (90.4)	348 (87.9)		–
	mean (SD)			MD (95%CI)
Gestational age at delivery (weeks)	38.8 ± 2.0	38.7 ± 1.8	0.63	−0.1 (−0.5; 0.3)
* Sensitivity analysis (per protocol 100%)* ^*b*^	*n* = 23	*n* = 396		
	*n* (%)			OR (95%CI)
Preterm birth			0.62	
< 37 weeks	2 (8.7)	48 (12.1)		0.7 (0.2;3.0)
≥ 37 weeks	21 (9.3)	348 (87.9)		–
	mean (SD)			MD (95%CI)
Gestational age at delivery (weeks)	38.8 ± 2.5	38.7 ± 1.8	0.87	−0.1 (−0.8;0.7)

^a^Description of these numbers was presented in the flowchart of the intervention. ^b^At least 48 sessions of the total exercise program. SD: standard deviation. OR: odds ratio. CI: confidence interval. *p > .05;* MD: mean difference

There were no significant differences in the incidence of GDM and preeclampsia (*p* > 0.05) between groups in the ITT and per protocol analyses (Table 3). In sensitivity analysis, there were 31 (7.6%) cases of GDM in the control group, while in the intervention group 1 (4.4%) case was identified (*p* = 0.56). There were 22 (5.4%) preeclampsia cases in the control group and no cases in the intervention group (*p* = 0.25).

**Table 3:** Gestational diabetes and pre-eclamspia in the exercise and control groups, PAMELA study

	Intervention (*n* = 205) ^ǂ^	Control (*n* = 407)^†^		
	*n* (%)		*p*	OR (95%CI)
*Intention-to-treat analysis*				
Gestational diabetes			0.93	
Yes	16 (7.8)	31 (7.6)		1.0 (0.6;1.9)
No	189 (92.2)	376 (92.4)		–
Pre-eclampsia			0.98	
Yes	11 (5.4)	22 (5.4)		1.0 (0.5;2.1)
No	194 (94.6)	385 (94.6)		–
*Per protocol analysis (> = 70% exercise sessions)*	*n*=85^a^	*n* = 407		
	*n* (%)			OR (95%CI)*P*
Gestational diabetes			0.85	
Yes	7 (8.2)	31 (7.6)		1.1 (0.5;2.6)
No	78 (91.8)	376 (92.4)		–
Pre-eclampsia			0.79	
Yes	4 (4.7)	22 (5.4)		0.9 (0.3;2.6)
No	81 (95.3)	385 (94.6)		–
Sensitivity analysis (per protocol 100%)^1^	*n* = 23	*n* = 407		
	*n* (%)			OR (95%CI)
Gestational diabetes			0.56	
Yes	1 (4.4)	31 (7.6)		0.6 (0.1-4.2)
No	22 (95.6)	376 (92.4)		–
Pre-eclampsia			0.25	
Yes	0	22 (5.4)		–
No	23 (100.0)	385 (94.6)		–

Data are expressed as number of cases in frequencies absolute and relative (*n*, %). *p* > .05; OR: odds ratio; CI: confidence interval. ^a^At least 48 sessions of the total exercise program

^ǂ^8 participants in the intervention group did not attend the perinatal follow-up

^†^19 participants in the control group did not attend the perinatal follow-up

^a^1 participant adherent to the PAMELA protocol did not attend the perinatal follow-up

Women in the intervention group gained less weight compared with those in the control group after 16 weeks of intervention for all three analyses (ITT, per protocol > = 70%, per protocol 100%), but this difference was not statistically significant (Table 4). The proportion of women in the exercise group gaining more weight than recommended by the IOM recommendations also did not differ from that in the control group in all three analyses.

**Table 4:** Maternal weight gain (kg) during pregnancy in the exercise and control groups, PAMELA study

*Intention-to-treat analysis*	Intervention (*n* = 155)^d^	Control (*n* = 320)^e^		
	mean (SD)		*p*	MD (95%CI)
Gestational body weight gain (kg)^a^	7.8 ± 3.5	8.4 ± 3.5	0.10	0.6 (−0.1;1.2)
	*n* = 205	*n* = 407		
Final gestational weight gain(kg)^b^	12.4 ± 5.7	12.9 ± 6.5	0.43	0.4 (−0.6; 0.8)
	*n* = 176	*n* = 351		
	*n* (%)			OR (95%CI)
IOM recommendations according pre-pregnancy BMI (kg/m^2^)^c^			0.79	
Below IOM recommendations	54 (30.7)	98 (27.9)		1.2 (0.7; 1.9)
Within IOM recommendations	55 (31.3)	117 (33.3)		–
Exceeded IOM recommendations	67 (38.0)	136 (38.8)		1.1 (0.7;1.6)
*Per protocol analysis (> = 70% exercise sessions)*	*n* = 84	*n* = 320		
	mean (SD)			MD (95%CI)
Gestational body weight gain (kg)^a^	7.6 ± 3.8	8.4 ± 3.5	0.10	0.7 (0.1;1.6)
	*n* = 85	*n* = 407	0.84	
Final gestational weight gain (kg)^b^	12.7 ± 5.7	12.9 ± 6.5		0.2 (−1.3; 1.7)
	*n* = 74	*n* = 351		
	*n* (%)			OR (95% CI)
IOM recommendations according pre-pregnancy BMI (kg/m^2^) ^c^			0.71	
Below IOM recommendations	22 (29.7)	98 (27.9)		1.3 (0.7; 2.4)
Within IOM recommendations	21 (28.4)	117 (33.3)		–
Exceeded IOM recommendations	31 (41.9)	136 (38.8)		1.3 (0.7; 2.3)
*Sensitivity analysis (100% of exercise sessions)* ^*c*^	*n* = 23	*n* = 320		
	mean (SD)			MD (95%CI)
Gestational body weight gain (kg) ^a^	7.6 ± 3.3	8.4 ± 3.5	0.31	0.8 (0.7; 2.2)
	*n* = 23	*n* = 407		
Final gestational weight gain (kg)^b^	11.7 ± 6.8	12.9 ± 6.5	0.42	1.1 (−1.6; 3.9)
	*n* = 21	*n* = 351		
	*n* (%)			OR (95%CI)
IOM recommendations according pre-pregnancy BMI (kg/m^2^) ^c^			0.13	
Below IOM recommendations	10 (47.6)	98 (27.9)		3.0 (0.9; 9.8)
Within IOM recommendations	4 (19.1)	117 (33.3)		–
Exceeded IOM recommendations	7 (38.8)	136 (33.3)		1.5 (0.4; 5.3)

^a^Difference between weight gain measured at baseline (16-20 weeks) and weight measured at the last visit to the clinic (32-36 weeks)

^b^Total gestational weight gain measured by self-reported and gathered at the hospital up to 48 h after delivery. ^c^Institute of Medicine guidelines for prenatal weight gain ^c^ At least 48 sessions of the total exercise program

^d^58 women in the intervention group and ^e^106 women in the control group did not attend the last visit at the clinic (32-36 weeks)

*p* > .05, *BMI* body mass index, *OR* odds ratio, *MD*, mean difference

There were no differences in the proportion of newborns small-for-gestational age and large-for-gestational age between intervention and control groups. The prevalence of newborns with low birth weight (< 2500 g) was not different between the intervention group (5.9%) and control group (4.9%) when considering ITT analyses (*p* = 0.90). Macrosomia (≥ 4000 g) was 4.4% and 5.2% in the intervention and control group, respectively. We also did not find statistically significant differences between the two groups in mean birth weight (*p* = 0.63), length (*p* = 0.33), and head circumference (*p* = 0.34) according to ITT analyses (Table 5). Similar results were found when these outcomes were evaluated by per protocol analysis.

**Table 5:** Offspring characteristics in the intervention and control group, PAMELA study

*Intention-to-treat analysis*	Intervention (*n* = 204)	Control (*n* = 407)		
	*n* (%)		*p*	OR (95%CI)
Small-for-gestational age (SGA)	8 (3.9)	22 (5.4)	0.42	0.7 (0.3; 1.6)
Large-for-gestational-age (LGA)	24 (11.8)	53 (13.0)	0.66	0.9 (0.5; 1.5)
Birth weight (g)			0.90	
< 2500	12 (5.9)	20 (4.9)		1.2 (0.6; 2.5)
≥ 4000	9 (4.4)	21 (5.2)		0.9 (0.4; 1.9)
	mean (SD)			MD (95%CI)
Birth weight (g)	3.234 ± 511	3.254 ± 467	0.63	19.7 (−61.5;100.9)
Birth length (cm)	48.2 ± 2.6	48.4 ± 2.2	0.33	0.2 (−0.2;0.6)
Head circumference (cm)	34.1 ± 1.8	34.2 ± 1.6	0.34	0.1 (−0.2;0.4)
*Per protocol analysis (> = 70% of exercise sessions)*	*n* = 85	*n* = 407		
	*n* (%)			OR (95%CI)
Small-for-gestational age (SGA)	4 (4.7)	22 (5.4)	0.79	0.86 (0.3; 2.6)
Large-for-gestational-age (LGA)	10 (11.8)	53 (13.0)	0.75	0.9 (0.4; 1.8)
Birth weight (g)			0.68	
< 2500	5 (5.9)	20 (4.9)		1.22 (0.44-3.35)
≥ 4000	5 (5.9)	21 (5.2)		1.16 (0.42-3.18)
	mean (SD)			MD (95%CI)
Birth weight (g)	3.300 ± 474	3.254 ± 467	0.41	−46.5 (−156.1; 63.1)
Birth length (cm)	48.6 ± 2.4	48.5 ± 2.2	0.52	−0.2 (−0.7; 0.4)
Head circumference (cm)	34.2 ± 1.6	34.2 ± 1.6	0.96	0.0 (−0.4;0.4)
*Sensitivity analysis (100% of exercise sessions)* ^*1*^	*n* = 23	*n* = 407		
	*n* (%)			OR (95%CI)
Small-for-gestational age (SGA)	2 (8.7)	22 (5.4)	0.50	1.7 (0.4; 7.6)
Large-for-gestational-age (LGA)	2. (8.7)	53 (13.0)	0.55	0.6 (0.1; 2.8)
Birth weight (g)			0.53	
< 2500	2 (8.7)	20 (4.9)		1.74 (0.38-7.96)
≥ 4000	–	21 (5.2)		–
	mean (SD)			MD (95%CI)
Birth weight (g)	3.244 ± 424	3.254 ± 467	0.62	0.5 (−145.8; 245.5)
Birth length (cm)	48.4 ± 2.0	48.5 ± 2.2	0.96	0.0 (−0.9;0.9)
Head circumference (cm)	34.0 ± 1.0	34.2 ± 1.6	0.54	0.2 (−0.5;0.9)

^ǂ^8 participants in the intervention group was not captured in the perinatal study and 1 had invalid data for offspring characteristics

^†^19 participants in the control group was not captured in the perinatal study

^a^1 participant adherent to the PAMELA protocol was not captured in the perinatal study

^1^At least 48 sessions of the total exercise program

*OR* odds ratio, *MD* mean difference, *p* > .05, C*I* confidence interval, *g* grams, *cm* centimeters

## Discussion

To the best of our knowledge, this is one of the first RCTs to apply a supervised exercise program evaluating a large number of maternal and neonatal outcomes within the same study in a middle income country. The present report indicates that supervised regular, moderate-to-vigorous exercise program performed three times/week did not support the benefits of exercise performed during pregnancy on maternal and newborn health outcomes evaluated.

The strengths of our study were the use of a RCT design, conducted by certified professionals in a supervised setting. The participants’ adherence to the exercise protocol was monitored both by the instructors and via recordings in a training diary. The intervention was planned according to the ACOG recommendations [2]. However, some limitations should be noted. First, besides several strategies to keep adherence to the exercise program (i.e., door-to-door transportation, fitness clothes to the intervention group, printed laboratory results from the blood and urine samples, and T-shirts of the trial for all participants), we had a higher number of dropouts in the intervention group and lower adherence to the protocol. Higher maternal age and schooling and not smoking during pregnancy were positively associated with adherence to the exercise intervention. Also, women with lower schooling and without a paid job during pregnancy were more likely to be lost to follow-up (data not show). However, the sample that was included in the ITT analysis did not present differences in baseline characteristics between intervention and control groups. The dropouts may have underpowered our analysis. Secondly, we did not record information about nutritional intake, although all women were exposed to the same standard care. Lack information on medical history of preeclampsia (as well timing in pregnancy) and gestational diabetes in prior pregnancy may be also consider limitations of our study. Preeclampsia was based on self-report and it might lead to misclassification. Thirdly, the timing of the intervention (lasting until 32-36 weeks) overlapped with the typical onset of preeclampsia and early preterm birth. However, we decided to finish the intervention at this time to prevent dropouts since involvement in physical activity declines in the final period of gestation [27–29]. Fourthly, our eligibility criteria may have been too rigorous which resulted in an extremely healthy population. Intragroup homogeneity may have made it difficult to find an effect of exercise among very healthy groups in a 16-week training window.

The effect of exercise during pregnancy on newborn’s outcomes is still unclear. Despite contrary evidence [30], pregnant women are often encouraged to decrease their levels of physical activity or even quit because of the belief that exercise may reduce placental circulation and, consequently, increase the risk of disorders such as miscarriages, preterm deliveries, and intrauterine growth retardation. However, results from 17 previous trials evaluating exercise during pregnancy and gestational age have showed no difference between exercise and control groups in mean of gestational age at delivery [12]. Similarly, our findings showed that regular exercise did not affect the mean gestational age when comparing the control and intervention groups. Although several cohort studies of LTPA suggest a reduction in the risk of preterm birth [31–33], we did not find differences between intervention and control groups.

This is one of very few RCTs investigating the effect of a supervised structured exercise program on preeclampsia. Previous meta-analyses have shown that exercise is a protective factor for hypertension and other cardiovascular diseases [34, 35]. Given that preeclampsia and cardiovascular disease share several risk factors, it has been hypothesized that physical activity may also protect against preeclampsia [8], but epidemiologic studies have not shown consistent results. According to the a recent meta-analysis [12], only three RCTs were conducted to evaluate the effect of exercise on development of preeclampsia. In our study, we did not find association between an exercise program during pregnancy and preeclampsia. Similar results were found in previous RCTs [36–38]. Aune et al. (2014) [8] conducted a systematic review and meta-analysis of seven cohort and four case–control studies, and found an inverse association between physical activity and preeclampsia. However, little robust evidence from randomized controlled trials is available to confirm these findings.

In terms of GDM, the results from previous RCTs are controversial. A recent review conducted by our group [12] showed a protective effect of exercise programs during pregnancy on the development of GDM when evaluating 11 RCTs, but the same was not observed in the meta-analysis of six RCTs conducted by Yin et al. (2014) [11]. The inverse association between exercise and development of GDM is biologically plausible. The main hypothesis is that exercise-induced improvements on glucose metabolism may be due to increases in GLUT4, direct effects on oxidative stress and endothelial function [39]. Also, exercise has an indirect and potentially more long-term role in glucose tolerance through changes in body composition [9]. However, in this trial there was no significant difference in the incidence of developing gestational diabetes between the intervention and control groups.

Most of the intervention studies that have evaluated the role of exercise in the prevention of gestational weight gain have found an inverse association between physical exercise during pregnancy and gestational weight gain [12]. Our results demonstrated that women who participated in the intervention gained on average 1 kg less than women in the standard care group, but this difference was not significant, probably because we did not have statistic power to find a difference for this variable. Moreover, we did not find differences in excessive gestational weight gain according to the IOM 2009 guidelines in both ITT and per protocol analyses. These findings contrast with the results of an intervention conducted by Ruiz et al. (2013) [40] that found women in the intervention group submitted to light- to moderate-intensity aerobic and resistance exercises were less likely to gain weight above the IOM recommendations compared with those in the standard care group. Given the negative consequences [41, 42] that have been associated between excessive gestational weight gain and maternal-child health outcomes, gestational weight management strategies should be considered high priority.

Regarding birth weight, a recent meta-analysis with 22 trials evaluated the effect of exercise interventions on birth weight [12]. No association was found regarding the effect of exercise on average birth weight. Our results support these findings. The clinical importance of a small reduction in mean birth weight is questionable, and it may be more relevant if maternal exercise primarily decreased the number of newborns with macrosomia, which may reduce the risk of prolonged labor and fetal hypoxia [43].

High compliance in intervention studies with pregnant women remains a challenge. Only 86 women (40.4%) reached our criterion of adherence to the protocol. Stafne et al. (2012) [37] found a similar result when reported that adherence to protocol (exercising 3 days per week or more at moderate to high intensity) in their study was 55% at follow-up at 36 weeks’ gestation. Non-adherence to prescribed behavior changes can substantially diminish the long-term benefits of health promotion programs. In our study, we had a high number of dropouts. One of the main reasons was medical advice to discontinue exercise. Previous studies have shown that pregnant women do seek advice about physical activity; however only 28.1% reported to be encouraged from health providers in prenatal care to physical activity practice [44]. Pregnant women whose health providers discussed exercise were more likely to report exercise during pregnancy, especially during late pregnancy [45]. Given that physical activity advice during prenatal care may be a predictor of exercise during pregnancy, these professionals have an essential role in the encouragement and support of these women to be physically active during pregnancy. It is essential that health professionals are conscious of current recommendations and benefits of physical activities during pregnancy. It is important to explore and understand all PAbarriers and facilitators when designing antenatal PA interventions in order to uncover the reasons for non-adherence and non-engagement with the behaviour, as well as determining what type of intervention would be acceptable [46].

## Conclusions

This study did not support the benefits of exercise performed during pregnancy on preeclampsia, weight gain and gestational diabetes. The results of this RCT showed that an exercise program did not find adverse impact on maternal-child health. However, the results of our study should be interpreted with caution given lack of statistical power and low compliance. Although the effectiveness of physical exercise programs on improving maternal and neonatal outcomes has been studied, the impact of physical activity on preeclampsia and birth weight is lacking. High-quality RCTs are still necessary to clarify the optimal frequency, type, duration and intensity of physical exercise required for beneficial health outcomes during pregnancy. Additional research is needed, in particular, to study the effects of physical exercise on newborn’s outcomes. This is an important area that should be explored further in future research. Studies on the effect of adherence strategies focusing in specific subgroups to enhance motivation for regular participation in exercise during pregnancy are also warranted.

## Additional files

Additional file 1: Table S1. Maternal characteristics at baseline for the participants included in the intention-to-treat analysis for the preterm birth analyses in the intervention group and control group; PAMELA study. (DOCX 39 kb)

Additional file 2: Table S2. Maternal characteristics at baseline for the participants included in the intention-to-treat analysis for the preterm birth in the intervention group and control group; PAMELA study. (DOCX 38 kb)

## Abbreviations

ACOG: American College of Obstetricians and Gynecologists
BMI: Body mass index
GDM: Gestational diabetes mellitus
IOM: Institute of Medicine
ITT: Intention-to-treat
LMP: Last menstrual period
LTPA: Leisure-time physical activity
PAMELA: Physical Activity for Mothers Enrolled in Longitudinal Analysis
RCT: Randomized controlled trial
WHO: World health organization
