# Supplementary material for: A randomized controlled trial of exercise during pregnancy on maternal and neonatal outcomes: results from the PAMELA study
Source: Int J Behav Nutr Phys Act. 2017 Dec 22;14:175. doi: 10.1186/s12966-017-0632-6 (PMC5741924; doi:10.1186/s12966-017-0632-6)
Supplement: Supplementary file 1 — Maternal characteristics at baseline for the participants included in the intention-to-treat analysis for the preterm birth analyses in the intervention group and control group; PAMELA study. (DOCX 39 kb) [file 12966_2017_632_MOESM1_ESM.docx]

**Additional file 1**

**Table S1** Maternal characteristics at baseline for the participants included in the intention-to-treat analysis for the preterm birth analyses in the intervention group and control group; PAMELA study

|  | **Intervention (n=198)** |  | **Control**  **(n=396)** | *p* |
| --- | --- | --- | --- | --- |
| ***Baseline measures***  ***(16-20 weeks gestation)*** |  |  |  |  |
| **Maternal age (years)** | 27.1 ±5.3 |  | 27.1 ±5.7 | 0.96 |
| **Gestational age** | 16.5 ±1.6 |  | 16.4 ±1.5 | 0.61 |
| **Weight (kg)** | 68.5 ±11.5 |  | 69.4 ±13.0 | 0.44 |
| **Height (cm)** | 161 ±6.5 |  | 161 ±6.0 | 0.33 |
| **Blood systolic pressure (mmHg)** | 111.9 ±10.2 |  | 111.8 ±10.4 | 0.96 |
| **Blood diastolic pressure (mmHg)** | 68.9 ±7.9 |  | 69.6 ±7.2 | 0.26 |
| **Proteinuria (mg/DL)^ǂ^** | 12.0 ±5.8 |  | 12.5 ±6.8 | 0.40 |
| **Fasting glycemia (mg/DL)** | 82.7±8.6 |  | 82.3±7.9 | 0.58 |
| ***Prenatal care measures***  ***(up to 20 weeks gestation)*** | **Intervention (n=184)** |  | **Control**  **(n= 370)** | *p* |
|  |  |  |  |  |
| **Schooling (years)** | 12.5 ±3.6 |  | 12.0 ±3.5 | 0.10 |
| **Pre-pregnancy body mass index (kg/m^2^)** | 25.1 ±3.8 |  | 25.1 ±4.2 | 0.90 |
| **Pre-pregnancy body mass index**  **(≥ 25 kg/m^2^)** | 75 (44.3) |  | 154 (45.2) | 0.87 |
| **Nulliparity** |  |  |  |  |
| Yes | 121 (65.8) |  | 247 (66.8) | 0.77 |
| **Skin color** |  |  |  |  |
| White | 139 (75.5) |  | 290 (78.6) | 0.10 |
| **Marital Status** |  |  |  |  |
| Living with a partner | 158 (85.9) |  | 319 (86.2) | 0.91 |
| **Smoking during pregnancy** |  |  |  |  |
| Yes | 10 (5.7) |  | 15 (4.4) | 0.51 |
| **Employment during pregnancy** |  |  |  |  |
| Yes | 105 (59.0) |  | 229 (64.0) | 0.26 |
| Data are expressed as means with standard deviation (SD) and n (%). *p* >.05  No statistically significant differences between groups. Group-mean differences according covariates were analyzed by the Student’s t-test (mean, SD) or chi-squared test (n, % ). | | | | |
