# Supplementary material for: A randomized controlled trial of exercise during pregnancy on maternal and neonatal outcomes: results from the PAMELA study
Source: Int J Behav Nutr Phys Act. 2017 Dec 22;14:175. doi: 10.1186/s12966-017-0632-6 (PMC5741924; doi:10.1186/s12966-017-0632-6)
Supplement: Supplementary file 2 — Maternal characteristics at baseline for the participants included in the intention-to-treat analysis for the preterm birth in the intervention group and control group; PAMELA study. (DOCX 38 kb) [file 12966_2017_632_MOESM2_ESM.docx]

**Additional file 2**

**Table S2** Maternal characteristics at baseline for the participants included in the intention-to-treat analysis for the preterm birth in the intervention group and control group; PAMELA study

|  | **Intervention group** | | | |
| --- | --- | --- | --- | --- |
|  | **Adherent (n=86)** |  | **Non-adherent (n=127)** | *p* |
|  |  |  |  |  |
| **Maternal age (years)** | 28.8 ±5.0 |  | 26.1 ±5.2 | **<0.001** |
| **Pre-pregnancy BMI (kg/m^2^)** | 25.0 ±3.7 |  | 25.2 ±4.0 | 0.66 |
| **Schooling (years)** | 13.7±3.3 |  | 11.5±3.7 | **<0.001** |
| **Pre-pregnancy BMI (n, %)^a^** |  |  |  | 0.49 |
| Under | 1 (1.3) |  | 3 (2.8) |  |
| Normal | 44 (58.7) |  | 52 (48.2) |  |
| Overweight | 22 (29.3) |  | 36 (33.3) |  |
| Obese | 8 (10.7) |  | 17 (15.7) |  |
| **Nulliparity** |  |  |  | 0.07 |
| Yes | 52 (63.4) |  | 72 (66.1) |  |
| **Skin color** |  |  |  | 0.40 |
| White | 61 (73.5) |  | 88 (75.9) |  |
| **Marital Status** |  |  |  | 0.97 |
| Living with a partner | 71 (85.5) |  | 99 (85.3) |  |
| **Smoking during pregnancy** |  |  |  |  |
| Yes | 2 (2.5) |  | 11 (10.0) | **0.04** |
| **Paid job during pregnancy** |  |  |  | 0.95 |
| Yes | 48 (58.5) |  | 61 (58.1) |  |
| Data are expressed as means with standard deviation (SD) and n. (%). *p* >.05  No statistically significant differences between groups. Group-mean differences according covariates were analyzed by the Student’s t-test (mean, SD) or chi-squared test (n, % ). ^a^ Variable with larger number of missing information (n=30) | | | | |
